# miTarget: microRNA target gene prediction using a support vector machine

**DOI:** 10.1186/1471-2105-7-411

**Published:** 2006-09-18

**Authors:** Sung-Kyu Kim, Jin-Wu Nam, Je-Keun Rhee, Wha-Jin Lee, Byoung-Tak Zhang

**Affiliations:** 1 Graduate Program in Bioinformatics, Seoul National University, Seoul, Korea; 2 Center for Bioinformation Technology (CBIT), Seoul National University, Seoul, Korea; 3 Biointelligence Laboratory, School of Computer Science and Engineering, Seoul National University, Seoul, Korea

## Abstract

**Background:**

MicroRNAs (miRNAs) are small noncoding RNAs, which play significant roles as posttranscriptional regulators. The functions of animal miRNAs are generally based on complementarity for their 5' components. Although several computational miRNA target-gene prediction methods have been proposed, they still have limitations in revealing actual target genes.

**Results:**

We implemented miTarget, a support vector machine (SVM) classifier for miRNA target gene prediction. It uses a radial basis function kernel as a similarity measure for SVM features, categorized by structural, thermodynamic, and position-based features. The latter features are introduced in this study for the first time and reflect the mechanism of miRNA binding. The SVM classifier produces high performance with a biologically relevant data set obtained from the literature, compared with previous tools. We predicted significant functions for human miR-1, miR-124a, and miR-373 using Gene Ontology (GO) analysis and revealed the importance of pairing at positions 4, 5, and 6 in the 5' region of a miRNA from a feature selection experiment. We also provide a web interface for the program.

**Conclusion:**

miTarget is a reliable miRNA target gene prediction tool and is a successful application of an SVM classifier. Compared with previous tools, its predictions are meaningful by GO analysis and its performance can be improved given more training examples.

## Background

MicroRNAs (miRNAs) are endogenous ~22 nucleotide noncoding RNAs, which act as posttranscriptional regulators in animals and plants. MiRNAs use two distinct posttranscriptional mechanisms to downregulate gene expression. They act by binding to the complementary sites on the 3' untranslated region (UTR) of the target gene to induce cleavage with near perfect complementarity or to repress productive translation [1-6]. They also facilitate deadenylation, which leads to rapid mRNA decay [7,8]. The choice between the translational inhibition and destruction is thought to be governed by the degree of mismatch between a miRNA and its target mRNA. The behaviors of miRNAs differ between animals and plants. Those of plants tend to show near perfect complementarity to their target messenger RNAs (mRNAs), but the miRNAs of animals usually have imperfect characteristics, including mismatches, gaps, G:U wobble pairs and others [9-15]. This makes it hard to find target animal mRNAs using only sequence complementarity. Nevertheless, strong sequence conservation observed in target mRNA sites and in miRNA sequences makes it possible to develop programs for the prediction of potential targets [16-18]. This evolutionarily meaningful evidence shows the importance of sequence preservation as a requirement for function. Particularly, no specific role has been explained for the 3' ends of miRNAs even though they tend to be evolutionarily conserved over their entire lengths.

To date, computational methods have been widely used for the prediction of miRNAs [16,19-21] and miRNA target genes [12,22-28]. Different approaches have been used for miRNA target predictions in plants and animals. For plant sequences, similarity-based approaches have shown high performance because complementarity is nearly perfect [22,25]. However, such approaches are not appropriate for animal genomes because of the imperfect nature of the miRNA:mRNA interaction. Studies for animal sequences have been based on both the complementarity to the 5' part of miRNAs and conserved motifs over species [12,23,24,26]. These can be implemented by a model containing weighted position features and comparative information to detect target mRNA sites and to reduce false positives. Scoring methods using dynamic programming [26,27,29] and a complementarity-based strategy. [23,28] are generally preferred to rank the prediction results. They have been quite successful for a few top-ranked results. However, the results are often limited by the conserved nature of the data set used.

In this article, we present a support vector machine (SVM) classifier to predict miRNA target genes. An SVM is one of the most popular machine learning algorithms and it has good performance in classification problems. Moreover, we collected training data from the literature to make a biologically relevant simulation. Generally, the efficiency and the reliability of a machine learning algorithm depend on choosing relevant data and specific features. Thus, a biologically relevant data set is as important as a good algorithm. An SVM builds a classifier directly from the data by investigating its characteristics. It does not require conservation information for classification, so it is free from the limitations described above. Our SVM classifier gave good results for predicting the targets of miRNAs.

## Implementation

### Biologically relevant data set

We collected our training data set from the literature. It contains 398 biologically meaningful examples, which are described in Table 1 and Supplementary Table 1 [see Additional file 1]. In the data collection step, we excluded examples that were not verified by wetlab experiments. In most miRNA function studies, miRNA target sites have often been predicted as putative ones based on complementarity, without experimental verification of precise target sites. These data may include both genuine and false binding sites. Consequently, we excluded all unconfirmed targets to improve the quality of our data set if the exact binding site could not be verified clearly. In addition, we double-checked the alignment of the examples in papers because illustrations and presented target sequences were often ambiguous. We checked the exact sequences with the miRNA sequences from the Rfam database [53] and with 3' UTR sequences from the Ensembl database [54].

**Table 1:** The training data set configuration.

Authors	Positive/negative (inferred)	Reference
Stark et al.	3/0	[12]
Johnston et al.	1/0	[50]
Nelson et al.	1/114(113)	[51]
Kiriakidou et al.	26/23	[27]
Vella et al.	0/57(50)	[13]
Doench et al.	29/15	[37]
Yekta et al.	2/0	[52]
Lai et al.	51/10	[48]
Brennecke et al.	39/27	[2]

The middle column contains the number of positive and negative examples gained from each reference. The number of inferred negative examples is indicated in parentheses.

The training data set gained directly from the literature contained 235 examples including 152 positives and 83 negatives. There were too few negative examples to build an effective classifier. We needed more negative data because these usually contribute to the specificity of a classifier much more significantly than positive data. Specificity is usually more important than sensitivity in genome analysis because slight decreases in specificity values can generate many false predictions because of the large size of genome sequences. However, we did not use randomly generated negative examples because such sequences often interact with miRNAs, as shown in the signal-to-noise ratio experiments of previous studies [23,30,31]. Instead, we inferred 163 negative examples as described below. Thus, the final size of the data set was 398 (152 positives, 246 negatives).

For the inferred negative examples, we noted that deletion of target sites on the target mRNA sequence can give a large number of negative examples. Thus, in one report [13], *let-7 *miRNA could not repress expression after deleting the target sites of *let-7 *miRNA on *lin-41*, and in another [4], *let-7 *miRNA was inactivated by knocking out the target sites on the gene for the cold shock protein LIN-28. That is, the remaining region on the *lin-41 *3' UTR will not now work with *let-7 *miRNA. This is the same for LIN-28. We conclude that if all the actual binding sites on *lin-41 *and LIN-28 are masked, then all the other remaining sites with favorable seed pairings are apposite as negative examples. In practice, we collected examples with more than 4-mer matches at their seed part and discarded the rest to improve the quality of the data set. As a result, we gained 163 inferred negative examples: 50 from *lin-41 *and 113 from LIN-28.

### Support vector machine

We used an SVM [32,33] to build a classifier discriminating the binding sites of a miRNA on the 3' UTR region of a gene. SVMs allow an implicit mapping of the sample vectors into a high-dimensional, non-linear feature space, in which the samples may be separated better using a similarity function between pairs of samples, called a kernel. To implement a kernel method, let us denote *S *= (*x*_1_,...,*x*_*n*_) as a set of miRNA target data to be trained. We suppose that each datum *x*_*i *_is an element of a set *X *of all possible target data. To design a data classification method, the data set *S *is then represented as the set of features, Φ(*S*) = (Φ(*x*_1_),..., Φ(*x*_*n*_)), where Φ(*x*) can be defined as a real-valued vector. The size of the vector is the number of features. This classification method is designed to process a set of pairwise comparisons of data *x*_*i *_and *x*_*j*_. It is represented by an *n *× *n *matrix of pairwise comparisons *k*_*i*,*j *_= *k*(*x*_*i*_,*x*_*j*_). The *n *× *n *matrix is used as input data of our kernel. In our study, a radial basis function (RBF) kernel is used:

***k***(***x***_*i*_, ***x***_*j*_) = exp(-*γ *||***x***_*i *_- ***x***_*j*_||^2^),     (1)

where the parameter *γ *determines the similarity level of the features so that the classifier becomes optimal.

SVMs are often believed to find an optimal hyperplane separating the training data. In practice, however, a separating hyperplane may not exist when a problem is very noisy or complex. To accommodate this case, slack variables *ξ*_*i *_≥ 0 for all *i *= 1,...,*n *are introduced to loosen the constraints as follows. [34]:

*y*_*i*_(⟨**w**,**x**_*i*_⟩ + *b*) ≥1 - *ξ*_*i *_for all *i *= 1,...,*n*.     (2)

A classifier that generalizes well is then obtained by adjusting both the classifier capacity ||**w**|| and the sum of the slacks ∑_*i *_*ξ*_*i*_. The latter can be shown to provide an upper bound on the number of training errors. Such a soft margin classifier can be realized by minimizing the following objective function:

$\frac{1}{2}{\left\|w\right\|}^{2}+C\sum_{i=1}^{n}\xi_{i}\;\left(3\right)$

subject to the constraints on *ξ*_*i *_and (2), where the constant *C *> 0 determines the trade-off between margin maximization and training error minimization. We implemented a modified version of SVMlight [35] to solve our problem.

### Parameter optimization and classifier evaluation

In this section, we describe the training and evaluation of classifiers and optimization of parameters. Before the evaluation of a classifier, a tool needs to train the classifier and optimize two SVM parameters, *C *and *γ*. We evaluated the classifier with a completely independent test data set. For this, we repeatedly performed three steps as follows. First we divided the data equally into training and test sets through random sampling (without replacement). Then we performed tenfold cross validation with the training data to train a classifier and to optimize parameters. Finally we evaluated the optimized SVM classifier with the remaining test data (which must be completely independent). We performed 10 repeated evaluations as above and averaged the results. For the adjustment of the two parameters, *C *and *γ*, we searched for a parameter set that maximized the accuracy of upper tenfold cross validation using:

$\underset{C,\gamma }{\arg\max}A(C,\gamma )\;\left(4\right)$

where *C *ranges from 1 to 200 in steps of 1.0 and *γ *ranges from 0.01 to 2.0 in steps of 0.01.

The discriminative power of our method can be described using receiver operating characteristic (ROC) analysis, which is a plot of the true positive rate against the false positive rate for the different possible cutoffs of a diagnostic test. ROC analysis reveals all possible trade-offs between sensitivity and specificity. For this, we measured the performance of classifiers across 24 cutoff points in the evaluation step (-4, -3, -2, -1.8, -1.6, -1.4, -1.2, -1, -0.8, -0.6, -0.4, -0.2, 0, 0.2, 0.4, 0.6, 0.8, 1, 1.2, 1.4, 1.6, 1.8, 2, 3). The ROC was plotted with the specificity and the sensitivity averaged from the results of 10 repeated evaluations.

### SVM features

**Figure 1:**
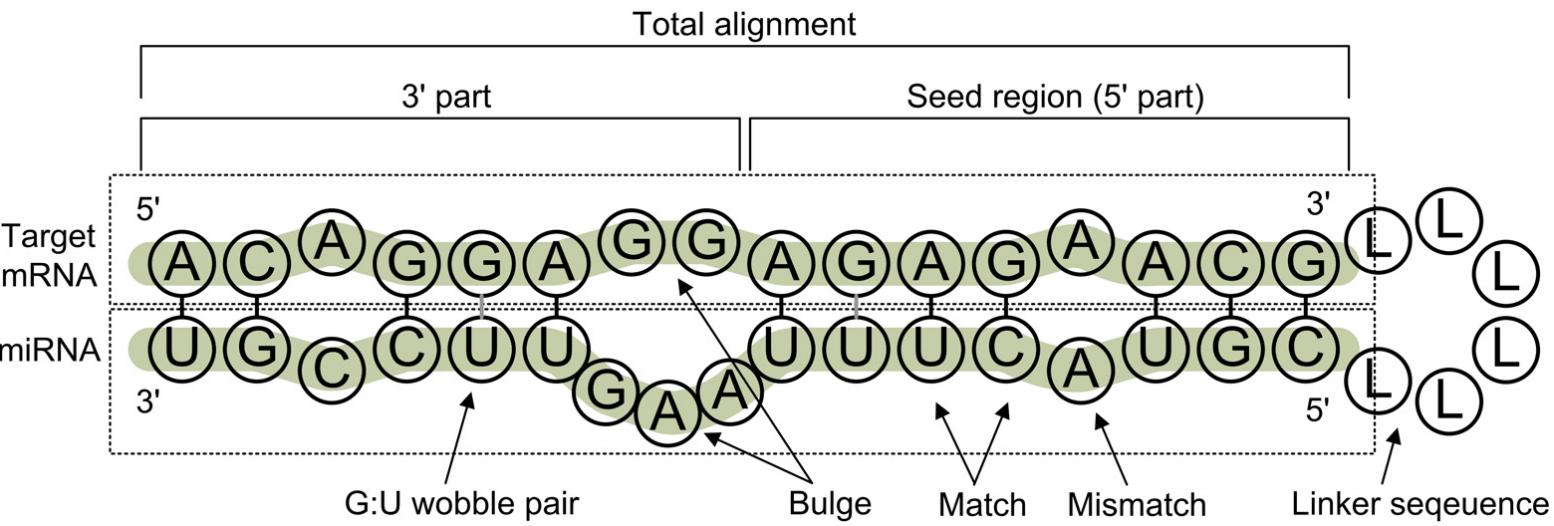
General scheme of miRNA:mRNA interactions.

**Figure 2:**
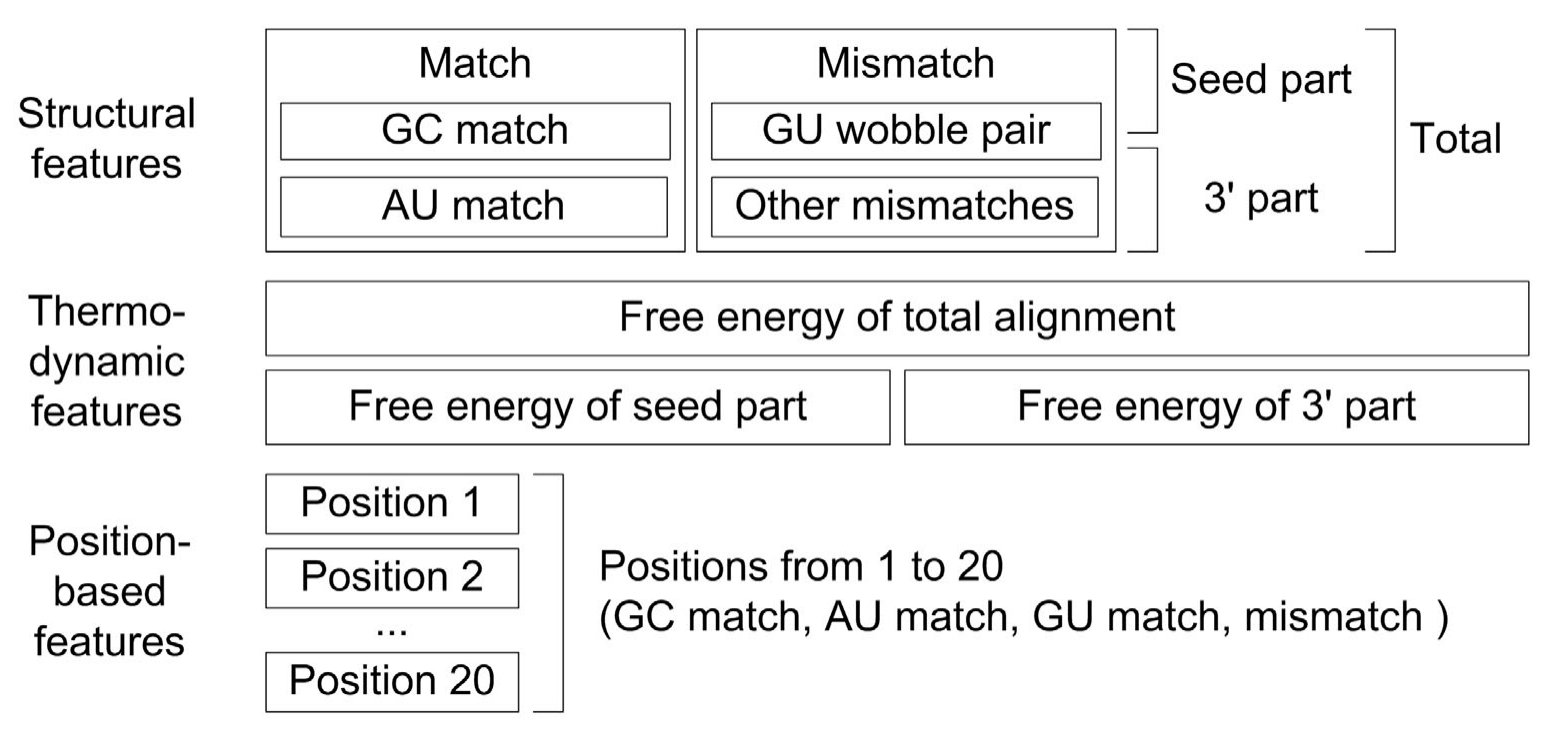
Three categories of SVM features.

SVM features are categorized into three elements: structural features, thermodynamic features and position-based features. Position-based features were introduced for the first time in this study, whereas the other structural and thermodynamic features have been used widely. We designed all features based on the RNA secondary structure prediction results produced by the RNAfold program in the Vienna RNA Package. [36]. The general scheme of miRNAs and their interactions with target mRNAs are illustrated in Figure 1. We used 41 different features, as shown in Figure 2. Structural and thermodynamic features had real values, and position-based features had nominal values. All values were normalized to have real values in the interval (0, 1).

The RNAfold program requires a single linear RNA sequence as input, so the 3' end of the target mRNA sequence and the 5' end of miRNA sequence are connected by a linker sequence, "LLLLLL". The "L" denotes that it is not an RNA nucleotide, thus it does not match with any nucleotide and so prevent mRNA and miRNA nucleotides from binding with sequence-specific linker sequences [12]. Thus, the RNAfold program produces an RNA secondary structure alignment with a linker sequence, exemplified in Figure 1. The positions in the alignment are numbered from the 5'-most position of the seed region. Alignments are extended until the 20th position and the rest positions are discarded.

For structural and thermodynamic features, we divided the secondary alignment into three parts consisting of the 5' part (seed part), the 3' part, and the total alignment as shown in Figures 1 and 2. Each count value of matches, mismatches, G:C matches, A:U matches, G:U matches, and other mismatches from the three parts was considered as a structural feature. The free energy values of the 5' part, the 3' part, and the total miRNA:mRNA alignment structure are thermodynamic features that are also calculated by RNAfold. Here, the sequence "AAAGGGLLLLLLCCCUUU" was used as a linker sequence to ensure that each part of the subsequence was paired. The sequences "AAAGGG" and "CCCUUU" were designed to prevent any unexpected alignment of the short matches. Although such linkers may change the original signal, the thermodynamic effect of the linker sequence will be the same for all short matches.

Position-based features are important because they imitate the shape and mechanism of the seed pairing. Doench et al. [37] and Brennecke et al. [2] focused on the sequence-specificity of miRNA:mRNA interaction. They found that a single point mutation could inhibit the miRNA's function depending on its position. In contrast to our earlier belief, their research revealed that examples with favorable thermodynamic free energy might not regulate expression. Therefore, we investigated the binding mechanism. Position-based features corresponded to point mutations in the above two experiments. Each position had one of the four nominal values consisting of a G:C match, an A:U match, a G:U match, and a mismatch. To make these values available for SVMlight, we translated them into decimal values from 1 to 4, respectively, and normalized them.

## Results

### Performance of the SVM classifier

**Figure 3:**
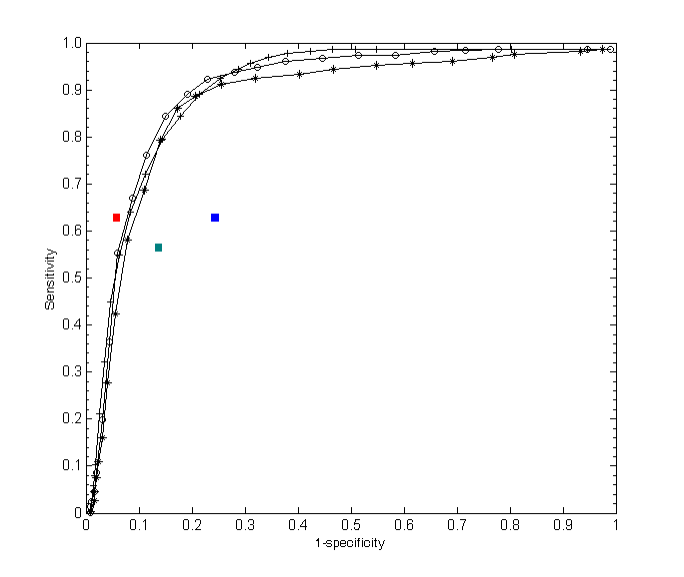
The ROC curves of classifiers created on three combinations of features: an entire set (circles), position-based features only (asterisks), and without position-based features (plus symbols). The red rectangle denotes the performance of TargetScan, the green one shows the performance of RNAhybrid, and the blue one shows the performance of miRanda.

We implemented miTarget, an SVM classifier, by modifying SVMlight for the effective analysis of miRNA:mRNA interactions. We analyzed the performance of miTarget using ROC curves and show the result in Figure 3. First, we tested the classifier with the complete feature set (circles). This gave an area under the ROC curve of 88.7%. Second, to investigate the effect of position-based features we evaluated the efficiency of the classifier after excluding them (plus signs). The ROC area was slightly decreased to 87.8%; sensitivity increased around the region of low specificity, but decreased around that of high specificity. Third, we tested it only with position-based features to evaluate the contribution of structural and thermodynamic features (asterisks). The ROC area was 84.6%, a decrease of 4.8%, and sensitivity and specificity were decreased across the range tested. Thus, the structural, thermodynamic, and position-based features improved the performance of the classifier synergistically and the position-based features enhanced its sensitivity.

### Supporting evidence from microarray data

Microarray experiments have been used widely for a variety of functional genomic studies. [38-41]. Through microarray experiments, Lim et al. [40] reported genes downregulated by miR-1, miR-124a, and miR-373, respectively. Although we could not use their data as a training example, because the specific binding sites have not been verified experimentally for each sequence, we could use them for a large list of miRNA target genes to verify our predictions. For this, we first retrieved the entire set of human 3' UTR sequences from the Ensembl database (20,008 unique sequences). This included 223 down-regulated genes out of 335 (Table 2). We then predicted target gene candidates for each miRNA using miTarget (Table 2). We aimed to match the target gene candidates to those genes downregulated by each miRNA in the microarray experiment. We found 75 shared genes (Table 2).

**Table 2:** Significance of miRNA target predictions based on miRNA microarray perturbation data.

	Down-regulated genes	With 3'UTR	miTarget	Common	*P*-values
miR-1	96	66	2,295	24	9.47E-08
miR-124a	174	117	3,048	36	1.02E-05
miR-373	65	40	2,964	15	2.62E-04

The downregulated gene # is the number of genes downregulated by overexpression of each miRNA, reported in Lim's paper [40] and "With 3'UTR" indicates the number of downregulated genes with a 3'UTR sequence. The "miTarget" is the number of target gene candidates predicted by miTarget; the "Common" means the number of candidates shared by "With 3'UTR". The *P*-value was calculated by hypergeometric testing.

We calculated probability (*P*)-value relative to hypergeometric distribution to test statistically significant enrichment of the downregulated genes among the target gene candidates using the following equation:

$\begin{array}{l} \begin{array}{cc} P{\left(x\right)}_{mir-1}=\frac{\left(\begin{array}{c} X\\ x \end{array}\right)\left(\begin{array}{c} N-X\\ n-x \end{array}\right)}{\left(\begin{array}{c} N\\ n \end{array}\right)}=\frac{\left(\begin{array}{c} 66\\ 24 \end{array}\right)\left(\begin{array}{c} 20008-66\\ 2295-24 \end{array}\right)}{\left(\begin{array}{c} 20008\\ 2295 \end{array}\right)} & P{\left(x\right)}_{mir-124a}=\frac{\left(\begin{array}{c} 117\\ 36 \end{array}\right)\left(\begin{array}{c} 20008-117\\ 3048-36 \end{array}\right)}{\left(\begin{array}{c} 20008\\ 3048 \end{array}\right)} \end{array}\\ P{\left(x\right)}_{mir-373}=\frac{\left(\begin{array}{c} 40\\ 15 \end{array}\right)\left(\begin{array}{c} 20008-40\\ 2964-15 \end{array}\right)}{\left(\begin{array}{c} 20008\\ 2964 \end{array}\right)} \end{array}$

where *X *denotes the number of downregulated genes (with 3' UTR sequences), *N *denotes the number of human 3' UTR sequences used for target prediction, *n *is the number of predicted target genes, and *x *is the number of downregulated genes matched by the predicted target genes. The *P*-values are shown in Table 2: the predicted targets were statistically significant.

In addition, we performed a significance test like that above to compare our method with a simple predictor, which searches for targets based on miRNA seed matches of positions 2–7, as a baseline. The results are summarized in Supplementary Table 4 [see Additional file 4]. In miR-124a and miR-373, excluding miR-1, miTarget was more significant than the seed match. Although target genes of miR-1 by miTarget were less enriched in downregulated genes, miTarget showed more robust target prediction of three miRNAs and the seed match showed a high number of false positives. Because several known miRNA:mRNA alignments have one or two mismatches in the seed region and some miRNAs mutated at the seed region are still functional, a simple approach based on the seed match may produce more false negatives.

It is necessary to emphasize that we note that some of the downregulated genes might be indirectly affected via other genes, so they may not be targets of these particular miRNAs. Also, some genuine targets may be repressed only translationally without being affected at mRNA level that is measured in the microarray experiments. Thus, although we cannot precisely measure it, the sensitivity of our classifier may be better than assumed here.

### Annotation using gene ontology (GO)

Using GO [42] to validate the target prediction is one of the most biologically relevant approaches for indicating the functional coherence of target genes [43]. It is achieved readily by searching for statistically significant GO terms.

**Figure 4:**
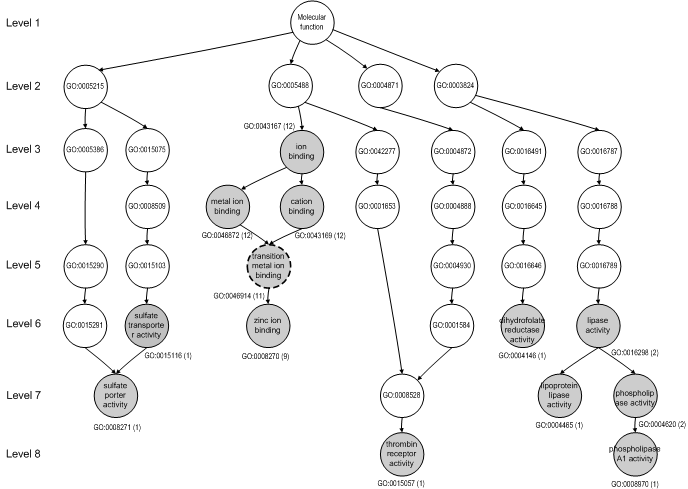
A subgraph of the GO-directed acyclic graph (DAG) to show functional relationships among the statistically significant GO terms for the target genes of miR-124a. The gray vertexes denote statistically significant GO terms based on a hypergeometric distribution. The numbers in brackets denote the numbers of genes annotated to the GO term. The dotted circle shows the best GO term.

To test if the target genes for each miRNA might be enriched functionally based on arbitrary GO terms, we performed GO annotation and significance analysis using GOstat [44, 55]. In the analysis, we observed terms associated significantly with the target genes (27 for miR-1, 26 for miR-124a, and 23 for miR-373) [see Additional file 2] included in the GO gene-association database (goa_human and Affymetrix HG_U95AV2 Human known genes) among the top 50 target genes. We used the default setting of GOstat. To find significantly overrepresented GO terms, GOstat calculates a *P*-value upon assuming hyper-geometric distribution of annotated GO terms. To control type I errors in multiple testing of GO terms, the *P*-values were adjusted to a False Discovery Rate (FDR) level of 0.1 [45]. For miR-1 and miR-124a, the most significant GO annotations were GO:0050517 (inositol hexakisphosphate kinase activity, adjusted *P *= 0.055) and GO:0046914 (transition metal ion binding, adjusted *P *= 0.0396) in the molecular function category, respectively. For miR-373, the best GO was GO:0016021 (integral to membrane, adjusted *P *= 0.000324) in the cellular component category. Figure 4 shows the statistically significant GO terms for miR-124a upon a subgraph of the Molecular Function category of GO. The graph was created by the function GOGraph of the GOstats R package. Supplementary Table 3 presents more details of the significant GO terms shown in Figure 4 [see Additional file 3].

**Table 3:** The top 15 contributing features.

Rank	Rank score	Feature
1	81.9	Position five
2	79.6	5' part free energy
3	79.1	Position six
4	78.9	Position four
5	78.9	AU matches at the 5' part
6	77.6	Mismatches at the 5' part
7	76.6	Matches at the 5' part
8	73.9	Total GU matches
9	73.4	Position seven
10	72.9	Position two
11	71.4	GU match at the 5' part
12	70.8	GU match at the 3' part
13	70.3	Total AU matches
14	68.8	Position three
15	68.6	Total free energy

### Comparison of random negative data sets

**Figure 5:**
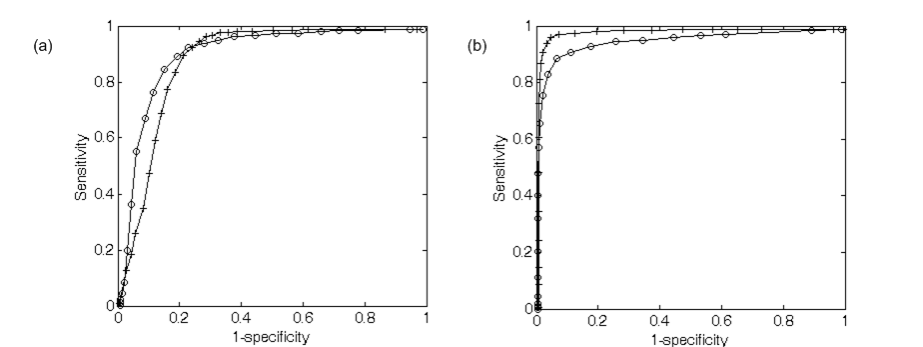
Comparisons between a random negative data set and an original negative data set. (a) The plots show the performance of the original (circle) and random (plus) classifiers on the original test data set. (b) The plots show the performance of the original (circle) and random (plus) classifiers on the random test data sets.

A previous study using random negative data sets produced a good numerical result [46]. Here, to compare the classifiers built with the original negative data and with the random negative data, we produced 246 random negative examples with the frequency used by Rajewsky et al. [28], and then constructed an original data set (152 positive data and 246 original negative data) and a random data set (152 positive data and 246 random negative data). We trained the classifiers with the original training data set (72 positive data and 123 original negative data) and with the random training data set (72 positive data and 123 random negative data), respectively, and then we performed an evaluation on the original test data set (remaining 72 positive data and 123 original negative data) and then on the random test data set (remaining 72 positive data and 123 random negative data) through the random sampling, as described in the section "Parameter optimization and classifier evaluation" (Figure 5a,b).

As can be seen in Figure 5a, the classifier built with the original data set showed a higher performance (ROC area: 88.7%) than the classifier built with the random data set (ROC area: 84.4%), as evaluated on the original test data set. This means that the classifier created on the original data set is more appropriate for genuine targets than the classifier created on the random data set. However, the random classifier (ROC area; 96.7%) showed a higher performance than the original classifier (ROC area; 93.3%) for random test data alone (Figure 5b).

The two results above indicate that a manually selected original data set is clearly important for the development of an efficient classifier. Although random negative data are widely used for machine learning algorithms, great care should be taken when using this approach. "Random" does not mean "negative", so the random data may contain real cases by chance, leading to relatively low sensitivity. In addition, such random data are often biologically infeasible, so they can be distinguished easily from positive data, which is why specificity is so high. Thus, we did not use random negative examples and used only actual examples from the literature, so that our data set was biologically relevant.

### Comparison with previous tools

There are several miRNA target gene prediction tools and each has its own merits. Lewis et al. [23] developed TargetScan to identify mammalian miRNA targets. It depends on a strong seed pairing mechanism and conservation among species, and gives an acceptable performance in wetlab experiments for validation. Enright et al. [24] implemented a dynamic-programming-based program, called miRanda, to identify targets for *Drosophila melanogaster*. They validated their result with wetlab experiments, but found a false positive rate of about 30%. Rehmsmeier et al. [29] improved existing RNA folding algorithms and presented RNAhybrid for prediction in the *Drosophila melanogaster *genome. By forcing seed matches on positions 2–7, it detected many previously known targets.

We performed a comparison on our miRNA:mRNA data set, and the ROC result is presented in Figure 3. Because the previous methods did not have cutoffs, we could not draw their ROC curves. Instead, we have indicated their performances as rectangles on the graph and compared specificities based on their sensitivity. Overall, miTarget gave a more stable performance than miRanda and RNAhybrid, but a slightly lower specificity (0.93) at a sensitivity of 0.63 than TargetScan (0.94). The higher specificity of TargetScan seems to arise from its strong constraint on the seed region. As it requires six continuous pairings on positions 2–7 of the seed region, this constraint made it predict many of the examples in our data set as negative. However, the limitation of TargetScan is that its best sensitivity was 0.63. Indeed, TargetScan seems to be rather conservative and is less flexible than our classifier, which can predict with optional accuracy across a broad range of specificity and sensitivity. Unlike other methods, our classifier is trainable and can be improved continuously if we can obtain more biologically relevant data.

### Contribution of each feature

We devised a feature selection method to investigate which might play a more dominant role in miRNA target regulation. Such methods are used to improve the performance of a classifier, to make it cost effective, and to help understand the problem. Our intention was to understand the hidden mechanism of miRNA function. We anticipated dominantly functioning features or noninformative features.

We used Weka software [47], and the features were evaluated using the OneR classifier and Ranker methods. The top 15 contributing features are shown in Table 3. Position-based features were ranked in half of the top 10 features, with position five in the lead. The continuous pairing of positions four, five, and six may be important for miRNA function, because they are ranked at fourth, first, and third, respectively. A G:U wobble pair also plays an important but maybe negative role; such a pair is known as a disturbing factor [4,13]. Therefore, G:U related features were ranked high. More than three G:U wobble pairs are believed to impair miRNA function [37], which is consistent with this result.

**Figure 6:**
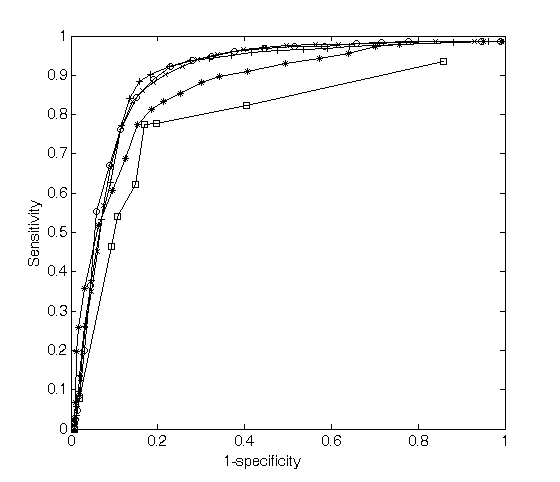
Changes in performance according to the numbers of features selected. The rectangle shows the ROC curve of the classifier created with the top feature, the asterisk (*) line is for the top five features, the plus symbol (+) line is for the top 10, the 'x' line is top 15, and the circle line is for the complete feature set.

We also investigated how many features were really contributing to the prediction results. We prepared data sets consisting of the top one, five, 10, 15, and all features, respectively. Each data set was trained and tested separately using the evaluation method described above, and the results are shown in Figure 6. The classifiers created on top five and top one features showed lower performances than others, but the classifiers built on top 10 and 15 features showed similar performances to the classifier created on the entire feature set. For sensitivity, there was a significant increase when we trained the classifier with up to the top 10 features. However, including the 31 other features could produce only 5% more sensitivity.

## Discussion

In this paper, we used an SVM classifier to predict miRNA target sites with biologically relevant data and measured its performance in various ways. We also investigated which features might contribute significantly to miRNA function. Our SVM classifier, called miTarget, performed well on our data set and produced significant results. Structural and thermodynamic features contributed to overall performance enhancement and position-based features increased specificity. miTarget also showed a stable performance compared with existing tools. Features on positions four, five and six seem to have more significant effects on seed pairing, according to the result of feature selection analysis. This is consistent with the general belief that the seed regions of paired structures should be stable. Moreover, out study suggests that individual positions in the seed region may unequally contribute to target recognition.

As mentioned above, high specificity is required in genome research because of the volume of data. For combinations of nucleotide pairs, the rates of match and mismatch were 0.25 and 0.75, respectively, and the expected specificity would be 0.75 with one position-based feature where a match is important: position five for example. One of the main limitations in miRNA target prediction for the human genome is the prevalence of long 3' UTR sequences compared with other species. The longer such sequences: the higher the false positive rate. In order to reduce the false positive rate, our classifier needs to be improved in specificity by introducing more inferred or experimental negative examples.

We verified our miRNA target-gene prediction results by the analysis of GO terms. We have shown the results for miR-1, miR-124a, and miR-373 and these are consistent with the general idea that miRNA targets are diverse in function [23]. To reveal more details of miRNA function, sophisticated wetlab experiments are essential for understanding the mechanisms of miRNA targeting.

According to a previous analysis [2], there should be three classes of miRNA target sites: canonical 5' dominant, seed dominant, and 3' compensatory. Two of these classes need to have strong complementarity on the seed. However, the 3' compensatory class needs to have only a moderate level of complementarity in the seed, while the 3' part is considerably matched. Our results failed to explain this. We found only one feature ranked at the 12th position, which was a G:U match at the 3' part, and almost all of the other features were about the 5' parts. This may be because the test was biased toward the effect of the miRNA seed. Recent studies have concentrated on the conservation of seed motifs. [48,49] and wetlab experiments are performed accordingly. Therefore, experiments on the 3' part are rarely done and it is hard to get appropriate data to investigate the effect of this region.

In addition, multiclass classification algorithms may be possible to explain this situation. If there really are three distinct classes in miRNA:mRNA pairing mechanisms, our binary classification approach is not an optimal solution. The lack of data is going to be another main limitation. Our data set is still small for standard machine learning approaches. However, for multiclass problems, the data set size should be much larger than the binary problem. Because machine learning algorithms often depend on the quality and amount of data set, many biologically verified high quality data are required.

## Conclusion

We constructed miTarget, an SVM classifier for miRNA target-gene prediction, and have shown its reliability in several ways. We collected a biologically relevant data set from the literature and designed new position-based features implying the manner of miRNA targeting. This predicted significant functions of human miRNA miR-1, miR-124a, and miR-373 by GO analysis. The feature selection experiment revealed that pairings at positions four, five and six are more important than other seed regions.

Nevertheless, there are still limitations in applying computer-based approaches. First, the actual mechanism of miRNA function remains unclear. Second, biologically relevant data are scarce. Third, "real" biological mechanisms can be species-specific. With more biologically relevant and unbiased data sets available, our SVM-based approach will be easily improved and create more reliable features reflecting the real actions of miRNAs.

## Availability and requirements

Project name: miTarget (microRNA target prediction)

Project home page: http://cbit.snu.ac.kr/~miTarget

Operating system(s): developed on Linux, Red Hat Enterprise Linux AS4

Programming language: Python, C

Other requirements: Vienna RNA package, SVMlight

License: none

## Abbreviations

miRNA, microRNAs.

mRNA, messenger RNA.

UTR, untranslated region.

SVM, Support Vector Machine.

RBF, radial basis function.

GO, gene ontology.

FDR, false discovery rate.

ROC, receiver operating characteristic.

DAG, directed acyclic graph.

## Authors' contributions

SKK, JWN, and WJL were involved in developing the ideas in this paper and writing this manuscript. SKK and JKR implemented the miTarget program and the web server. SKK and JWN performed the computational experiments and the follow up experiments. JWN analyzed the results including Gene Ontology methods. BTZ supervised the whole procedure and prepared this manuscript. All authors read and approved the final manuscript.

## Supplementary Material

Additional File 1 **Source of the training examples**. Supplementary Table 1. The table contains information about the papers from which the training data set was collected. The paper's title, author, gene name, and the corresponding miRNA name are listed. The numbers of examples for a pair are not shown because most of them are studies with several experimental mutations in one gene and its miRNA pair. Click here for file

Additional File 4 **Details of the statistical significance of seed match approach**. Supplementary Table 4. This table describes the significance of seed match approach upon miRNA microarray perturbation data using hypergeometric test. It is compared to the result of Table 2. Click here for file

Additional File 2 **The target gene list for GO analysis**. Supplementary Table 2. This table lists the targets among the top 50 target genes in the gene database. The genes were used for GO analysis for miR-1 (Supplementary Table 2-1), miR-124a (Supplementary Table 2-2), and miR-373(Supplementary Table 2-3). Click here for file

Additional File 3 **Details of the statistically significant GO terms**. Supplementary Table 3. This table lists statistically significant GO terms in the prediction results for miR-1 (Supplementary Table 3-1), miR-124a (Supplementary Table 3-2), and miR-373 (Supplementary Table 3-3). Click here for file
