# Supplementary material for: miTarget: microRNA target gene prediction using a support vector machine
Source: BMC Bioinformatics. 2006 Sep 18;7:411. doi: 10.1186/1471-2105-7-411 (PMC1594580; doi:10.1186/1471-2105-7-411)
Supplement: Additional File 4 — Details of the statistical significance of seed match approach. Supplementary Table 4. This table describes the significance of seed match approach upon miRNA microarray perturbation data using hypergeometric test. It is compared to the result of Table 2. [file 1471-2105-7-411-S4.doc]

Supplementary Table 4. Statistical significance of target prediction based on seed matches. The targets matched at seed positions 2-7 are identified by examining the miRNA:3’UTR alignment structure, folded by RNAfold. The *P-*values were estimated using hypergeometric testing as follows:

$$P(x)_{mir-1}=\frac{\left(\begin{matrix} 66 \\ 53 \end{matrix}\right)\left(\begin{matrix} 20008-66 \\ 5034-53 \end{matrix}\right)}{\left(\begin{matrix} 20008 \\ 5034 \end{matrix}\right)}$$

$$P(x)_{mir-124a}=\frac{\left(\begin{matrix} 117 \\ 72 \end{matrix}\right)\left(\begin{matrix} 20008-117 \\ 9238-72 \end{matrix}\right)}{\left(\begin{matrix} 20008 \\ 9238 \end{matrix}\right)}$$

$$P(x)_{mir-373}=\frac{\left(\begin{matrix} 40 \\ 25 \end{matrix}\right)\left(\begin{matrix} 20008-40 \\ 8831-25 \end{matrix}\right)}{\left(\begin{matrix} 20008 \\ 8831 \end{matrix}\right)}$$

“Downregulated genes” are the number of genes downregulated by overexpression of each miRNA, as reported by Lim [40] and “With 3’UTR” indicates the number of downregulated genes with a 3’UTR sequence. The “Seed match” is the number of target gene candidates matched by seed positions 2-7; the column “Common” shows the number of candidates shared by “With 3’UTR”. The P value was calculated by hypergeometric testing.

|  | Down-regulated genes | With 3'UTR | Seed match | Common | *P*-value |
| --- | --- | --- | --- | --- | --- |
| miR-1 | 96 | 66 | 5,034 | 53 | 6.87E-21 |
| miR-124a | 174 | 117 | 9,238 | 72 | 2.83E-04 |
| miR-373 | 65 | 40 | 8,831 | 25 | 8.50E-03 |
